# LncRNA LUCAT1 contributes to cell proliferation and migration in human pancreatic ductal adenocarcinoma via sponging miR‐539

**DOI:** 10.1002/cam4.2724

**Published:** 2019-12-02

**Authors:** Yongjun Nai, Chao Pan, Xueteng Hu, Yong Ma

**Affiliations:** ^1^ Department of General Surgery Nanjing First Hospital Nanjing Medical University Nanjing China; ^2^ The First Clinical Medical School Nanjing Medical University Nanjing China

**Keywords:** cell invasion, cell proliferation, lncRNA LUCAT1, miR‐539, pancreatic ductal adenocarcinoma

## Abstract

Pancreatic ductal adenocarcinoma is one of the most aggressive and dreadful malignancies worldwide. Long noncoding RNAs (lncRNAs) have emerged as vital regulators in the development of human malignancies and other disorders. This study aimed to characterize the role of lncRNA lung cancer‐associated transcript 1 (lncRNA LUCAT1), a novel cancer‐related lncRNA, in human PDAC. Here we initially analyzed the expression patterns of lncRNA LUCAT1 and evaluated its clinical significance. The qRT‐PCR analysis and in situ hybridization staining showed that lncRNA LUCAT1 expression was significantly increased in tumorous tissues compared with adjacent normal tissues. Additionally, we found that increased lncRNA LUCAT1 expression was linked to larger tumor size and lymphatic invasion. Consistently, lncRNA LUCAT1 was remarkably up‐regulated in PDAC cell lines. To better understand the biological role of lncRNA LUCAT1, we evaluated the effects of lncRNA LUCAT1 knockdown on PDAC cell proliferation, cell cycle progression, migration, and invasion using MTT assays, flow cytometry, Transwell migration, and invasion assays, respectively. Functional studies demonstrated that lncRNA LUCAT1 knockdown dramatically suppressed PDAC cell proliferation, induced cell cycle arrest and inhibited cell migration and invasion. Tumor xenograft in vivo assays displayed that lncRNA LUCAT1 inhibited tumorigenecity of PDAC cells. Mechanistic studies uncovered that lncRNA LUCAT1 acted as a molecular sponge of miR‐539 and that miR‐539 mediated the effects of lncRNA LUCAT1 on PDAC cell proliferation, cell cycle progression, and motility. Collectively, our findings may offer some novel insights into understanding lncRNA LUCAT1 in PDAC.

## Graphical abstract

In this study, we aimed to characterize the biological role of long noncoding RNA lung cancer‐associated transcript 1 (lncRNA LUCAT1), a novel tumor‐associated lncRNA, in human PDAC. Here we revealed the carcinogenic role of lncRNA LUCAT1 in PDAC. Furthermore, our data indicate that lncRNA LUCAT1 facilitates PDAC cell proliferation and invasion via sponging miR‐539, thereby offering some novel insights into understanding PDAC development.

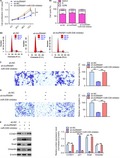

## 1 INTRODUCTION

Pancreatic cancer is one of the most common gastrointestinal malignancies and ranks as the fourth leading cause of cancer‐related mortality around the world.^1^, ^2^, ^3^ It was reported that about 350 000 people were diagnosed annually with pancreatic cancer worldwide.^4^, ^5^ It was estimated that around 50 000 new pancreatic cancer cases occurred annually in the United States and the 5‐year survival rate was less than 5%.^6^, ^7^ Pancreatic ductal adenocarcinoma (PDAC) is the most prevalent pathological type of pancreatic cancer and accounts for approximately 95% of all pancreatic cancer cases.^8^, ^9^ Nowadays, surgical resection, chemotherapy, and combination therapy remain the mainstay for treating PDAC.^10^, ^11^ In spite of the fact that great advances have been made in the diagnosis and treatment of PDAC, the mortality rate is still pretty high. Therefore, there is a pressing need to gain a comprehensive understanding of molecular mechanisms underlying PDAC malignant progression and develop more effective therapeutic alternatives for patients. In recent decades, molecular target therapy is regarded as a promising candidate therapeutic strategy against human malignancies.

Long noncoding RNAs (lncRNAs) are a novel and large group of non‐protein‐coding RNA transcripts longer than 200 nucleotides, which are identified to participate in diverse biological processes.^12^, ^13^, ^14^ A growing number of evidence has revealed that lncRNAs serve as pivotal regulators in the progression of human malignancies and other disorders.^15^, ^16^, ^17^ Long noncoding RNA lung cancer‐associated transcript 1 (lncRNA LUCAT1) is transcribed from a region on human chromosome 5.^18^ LncRNA LUCAT1, also termed as smoke and cancer‐associated lncRNA 1 (SCAL1), was first reported to be implicated in smoking‐related lung cancer.^19^, ^20^ Furthermore, recent studies have also demonstrated that lncRNA LUCAT1 is dysregulated and involved in multiple types of human malignant tumors, such as osteosarcoma,^21^ clear cell renal cell carcinoma,^22^ ovarian cancer,^23^ and esophageal squamous cell carcinoma.^24^ Nonetheless, the biological role of lncRNA LUCAT1 in PDAC remains largely unclear.

In this work, we aimed to characterize the biological role of lncRNA LUCAT1 in PDAC and clarify the possible molecular mechanism involved. Here our findings demonstrated a significant increase of lncRNA LUCAT1 expression both in PDAC tissue samples and cell lines. Moreover our results revealed that lncRNA LUCAT1 acted as miR‐539 sponge to facilitate PDAC cell proliferation, cell cycle progression, migration and invasion. The current study may provide some novel insights into understanding the biological functions of lncRNA LUCAT1 in human PDAC.

## 2 MATERIALS AND METHODS

### 2.1 Patients and tissue samples

This study was approved by the Institutional Review Board of Nanjing Medical University of China. All the participants provided written informed consents. Tumorous tissue samples and adjacent normal tissue samples were collected from 60 patients who underwent surgical resection between June 2010 and December 2017. All the clinical tissue samples were immediately stored at −80°C for further use.

### 2.2 RNA isolation and qRT‐PCR analysis

Total RNA was isolated from clinical tissue samples and culturing cells using the TRIzol reagents (Invitrogen) following the manufacturer's protocols. Complementary DNA (cDNA) was synthesized using the M‐MLV Reverse Transcriptase (Invitrogen) according to the manufacturer's instructions. qRT‐PCR was performed using SYBR Premix Ex Taq^™^ II Kit (Takara) on the ABI Prism 7500 Real‐Time PCR System (Applied Biosystems) in accordance with the manufacturer's protocols. β‐actin was used as an internal control for lncRNA LUCAT1. For miRNA expression analysis, qRT‐PCR was conducted using the TaqMan microRNA assays (Applied Biosystems) and U6 was used to normalize the expression of miRNA. The sequence of the specific primers were listed as followed: for lncRNA LUCAT1, 5′‐AGCTCCACCTCCCGGGTTCACG‐3′ (forward) and 5′‐CGTGAACCCGGGAGGTGGAGCT‐3′ (reverse); for β‐actin, 5′‐ACTGGAACGGTGAAGGTGAC‐3′ (forward) and 5′‐AGAGAAGTGGGGTGGCTTTT‐3′ (reverse); for miR‐539, 5′‐TATGATGAATCATACAAGGAC‐′3 (forward) and 5′‐GTCCTTGTATGATTCATCATA‐′3 (reverse); for U6, 5′‐CTCGCTTCGGCAGCACA‐3′ (forward) and 5′‐AACGCTTCACGAATTTGCGT‐3′ (reverse). Relative expression level was calculated using the 2^−△△Ct^ methods.

### 2.3 In situ hybridization

In situ hybridization was performed as described previously. Briefly, 5‐μm‐thick paraffin sections were deparaffinized and rehydrated, followed by treatment with Triton X‐100 to enhance probe penetration. The slides were washed using PBS three times and fixed again in 4% paraformaldehyde. After being washed three times with PBS, the slides were pre‐hybridized at 40°C for 4 hour. Subsequently, the sections were hybridized with digoxin‐labeled RNA oligonucleotide probe at 40°C overnight. Following being washed with different concentrations of saline sodium citrate at 50°C, the slides were blocked at 37°C for 1 hour and then incubated with anti‐digoxigenin‐alkaline antibody conjugated with phosphatase (Roche Diagnostics) at 4°C overnight. At the end, the sections were stained using NBT/BCIP mixture (Roche) and observed under a light microscope (Olympus).

### 2.4 Cell culture and transfection

PDAC cell lines (BxPC‐3, Capan‐2, AsPC‐1, and PANC‐1) and the immortalized pancreatic duct epithelial cell line HPDE6c7 were purchased from American Type Culture Collection and cultured in RPMI1640 medium (Gibco) containing 10% fetal bovine serum (FBS) at 37°C in a humified 5% carbon dioxide atmosphere.

Cell transfection was performed using the Lipofectamine 2000 (Invitrogen) according to the manufacturer's instructions. Short hairpin RNAs specifically targeting lncRNA LUCAT1 were designed and synthesized by GenePharma. The sequences of sh‐lncRNA#1 and sh‐lncRNA#2 were listed as followed: for sh‐lncRNA#1, 5′‐CACCTGTCCCTCAGTGTTCTACTCGAAAGTAGAACACTGAGGGACAGC‐3′ (sense) and 5′‐AAAAGCTGTCCCTCAGTGTTCTACTTTCGAGTAGAACACTGAGGGACAGC‐3′ (anti‐sense); for sh‐lncRNA#2, 5′‐CACCGTCCCTCAGTGTTCTACTTCTCGAAAGAAGTAGAACACTGAGGGAC‐3′ (sense) and 5′‐AAAAGTCCCTCAGTGTTCTACTTCTTTCGAGAAGTAGAACATGAGGGAC‐′3 (anti‐sense). sh‐lncRNA#1 and sh‐lncRNA#2 were inserted into pLKO.1 plamids (Sigma‐Aldrich). Transfection efficiency was determined using qRT‐PCR analysis at 48 hour post‐transfection.

### 2.5 MTT assays for cell proliferation

Cell proliferation was detected using MTT assays according to the manufacturer's instructions. Approximately 4 × 10^3^ cells/well were seeded into 96‐well plates and incubated at 37°C. Subsequently, 10 μL of MTT solution (5 mg/mL) was added into each well at different time points. Following 4 hours’ incubation with MTT solution, 200 μL of DMSO solution was added into each well to dissolve the precipitates and the absorbance value was measured at 560 nm using a micro‐plate reader (Bio‐Tek Instruments).

### 2.6 Flow cytometry analysis for cell cycle

Flow cytometry was applied to evaluate cell cycle progression of tumor cells as described previously.^25^ Briefly, 2 × 10^5^ cells were placed into 6‐well plates and incubated for 48 hours. Subsequently, cells were collected, washed three times with ice‐cold PBS, fixed with ice‐cold 70% ethanol overnight and stained using propidium iodide. Finally, cell cycle was evaluated using the FACSVerse flow cytometer (Becton Dickinson). All the experiments were performed in triplicates.

### 2.7 Transwell migration and invasion assays

Migration and invasion capabilities of tumor cells were evaluated using Transwell migration and invasion assays, respectively. Migration and invasion assays were performed using Transwell chambers (pore size of 8 μm; Corning). For the migration assays, 200 μL of cell suspension (4 × 10^4^ cells) was added into the upper chamber filled with serum‐free medium, and the cells were incubated for 24 hour. Then the cells in the upper chamber were disposed of, and the migrated cells in the lower chamber containing complete medium were fixed and stained using 0.1% crystal violet. For the invasion assays, the upper chamber was pre‐coated with Matrigel layer. 4 × 10^4^ cells were seeded in the upper chamber containing serum‐free medium, while complete medium with 10% FBS was added to the lower chamber. At 48 hour post‐inoculation, the cells remaining in the upper chamber were removed, and the cells invading into the lower chamber were fixed and stained using crystal violet dye. The stained cells were observed under an inverted microscope (Olympus) and counted in five random fields at a magnification of 400×.

### 2.8 Western blotting assays

Proteins were extracted from clinical tissue samples and culturing cells using the RIPA buffer supplemented with proteinase inhibitor cocktail (Pierce Biotechnology) according to the manufacturer's protocols. Protein concentrations were determined using the BCA Protein Assay Kit (Pierce). Extracted proteins were isolated using 10% SDS‐polyacrylamide gel electrophoresis and then transferred onto the PVDF membranes, followed by 5% nonfat milk blocking. Afterward, the membranes were probed using primary antibodies. The primary antibodies were listed as followed: anti‐CCND1 (1:1000, ab134175; Abcam), anti‐p21 (1:1000, #2947; CST), anti‐Snail (1:1000, ab180714; Abcam), anti‐Vimentin (1:1000, ab8979; Abcam), and anti‐β‐actin (1:500, ab20272; Abcam). Following incubation with HRP‐conjugated secondary antibody, the blots were visualized using the ECL detection system (Amersham).

### 2.9 Tumor xenograft model assays

This animal study was approved by the Animal Care and Use Committee of Jining No.1 People's Hospital. BALB/c nude mice (male, aged 4–6 weeks) were purchased from the Shanghai LAC laboratory Animal Co. Ltd. The sh‐lncRNA#1‐treated PANC‐1 cells (1 × 10^7^ cells in 100 μL) were subcutaneously inoculated into the flanks of the nude mice (n = 3). Tumor volumes were measured and recorded every 7 days. At day 35 post‐inoculation, all the nude mice were sacrificed, then the tumors were removed and weighed.

### 2.10 Immunohistochemical analysis

Immunohistochemical staining was used to visualize the expression of Ki67 and Snail proteins. In brief, paraffin sections at 5 μm thickness were prepared using clinical tissue samples. Then the sections were deparaffiinized in 100% xylene and rehydrated using gradient ethanol solutions and water. Antigen retrieval was performed in sodium citrate buffer (10 mmol L^−1^, pH 6.0) at 100°C for 10 minutes. Sections were stained using the primary antibodies for 1 hour at room temperature, followed by incubation of horseradish peroxidase‐conjugated secondary antibody. DAB was used as chromogen to visualize Ki‐67‐ and Snail‐positive staining.

### 2.11 RNA immunoprecipitation (RIP)

RIP assays were conducted using the EZ‐magna RNA Immunoprecipitation Kit (Millipore) in accordance with the manufacturer's protocols. In brief, cells were collected and lysed using RIP lysis buffer. The whole‐cell extracts were then incubated with RIP buffer containing magnetic beads conjugated to anti‐AGO2 or control IgG. The beads were washed using washing buffer, followed by addition of proteinase K to digest the proteins. Subsequently, the immunoprecipitated RNAs were isolated and subjected to qRT‐PCR analysis.

### 2.12 Luciferase reporter assays

The sequences of wild‐type lncRNA LUCAT1 and its corresponding mutant lncRNA LUCAT1 were synthesized by Sangon Bio‐technologies. Then the sequences were cloned into the pmirGLO plasmids (Promega) carrying luciferase reporter fragments to generate wild‐type lncRNA LUCAT1 reporter vectors or mutant lncRNA LUCAT1 reporter vectors. The reporter vectors were subsequently co‐transfected with miR‐539 mimics or negative control mimics into HEK293T cells using the Lipofectamine 2000 reagent (Invitrogen) according to the manufacturer's instructions. At 48 hour post‐transfection, the luciferase activity was monitored using the Dual‐Luciferase Reporter System (Promega) and the relative luciferase activity was normalized to *Renilla* luciferase reporter activity. All the experiments were performed in triplicates.

### 2.13 Statistical analysis

All the data were presented as the mean ± standard deviation. The SPSS 18.0 software (SPSS Inc) was used to perform statistical analysis. Two group comparison was conducted using a two‐tailed Student's *t*‐test. Multiple group comparison was carried out using one‐way ANOVA. The correlation between lncRNA LUCAT1 expression and clinicopathological characteristics was assessed via Fisher's exact test. *P* < .05 was considered statistically significant.

## 3 RESULTS

### 3.1 LncRNA LUCAT1 is significantly up‐regulated in PDAC tissues and cell lines

**Figure 1:**
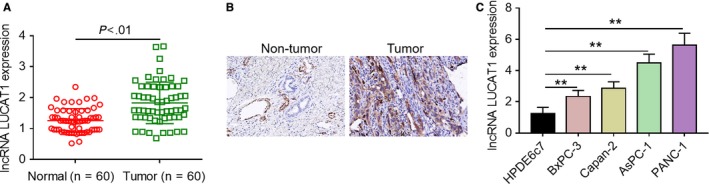
LncRNA LUCAT1 is significantly up‐regulated in PDAC tissues and cell lines. (A) Relative expression levels of lncRNA LUCAT1 were determined using qRT‐PCR analysis in tumorous tissue samples and adjacent normal tissue samples. (B) ISH staining was carried out to characterize the expression patterns of lncRNA LUCAT1 in cancerous tissue samples and matched normal tissue samples. (C) Relative expression levels of lncRNA LUCAT1 were examined via qRT‐PCR analysis in the immortalized pancreatic duct epithelial cell line HPDE6c7 and four human PDAC cell lines and. **P < *.*01. lncRNA LUCAT1, long non‐coding RNA lung cancer associated transcript 1; qRT‐PCR, quantitative real‐time polymerase chain reaction; ISH, in situ hybridization; PDAC, pancreatic ductal adenocarcinoma

To investigate the biological role of lncRNA LUCAT1 in human PDAC, qRT‐PCR analysis was firstly conducted to determine its expression levels in PDAC tissue samples and adjacent normal tissues samples. As shown in Figure 1A, lncRNA LUCAT1 expression was significantly increased in tumorous tissues compared with nontumorous tissues. Consistently, ISH staining demonstrated that higher lncRNA LUCAT1 expression was observed in cancerous tissues in comparison with normal tissues (Figure 1B). In addition, we examined the expression patterns of lncRNA LUCAT1 in four PDAC cell lines using qRT‐PCR analysis. As presented in Figure 1C, lncRNA LUCAT1 was highly expressed in four PDAC cell lines compared with the immortalized pancreatic duct epithelial cell line HPDE6c7. To evaluate the clinical significance of lncRNA LUCAT1, we analyzed the relationship between its expression levels in cancerous tissues and clinicopathological parameters. Tumorous tissues were classified into two groups, high lncRNA LUCAT1 expression group and low lncRNA LUCAT1 expression group, according to the median value of its expression levels. As summarized in Table 1, Fisher's exact test demonstrated that high lncRNA LUCAT1 expression was linked to larger tumor size and lymphatic invasion; moreover, no significant correlation was observed between lncRNA LUCAT1 expression and other clinicopathological characteristics. Taken together, these findings indicate that lncRNA LUCAT1 is significantly up‐regulated in both PDAC tissues and cell lines, and imply that increased lncRNA LUCAT1 expression may be associated with malignant progression of PDAC.

**Table 1:** Relationship between lncRNA LUCAT1 expression and clinicopathological features of PDAC patients

Characteristics	No. of cases	Low (n = 31)	High (n = 29)	*P* value
Gender				
Female	28	14	14	0.809
Male	32	17	15	
Age (years)				
<60	31	16	15	0.993
≧60	29	15	14	
CEA				
Positive	30	17	13	0.438
Negative	30	14	16	
Tumor size				
<4 cm	32	22	10	0.005*
≧4 cm	28	9	19	
Lymphatic invasion				
Yes	33	13	20	0.003*
No	27	18	9	

Abbreviation: CEA, carcinoembryonic antigen.

* *P* < .05.

### 3.2 LncRNA LUCAT1 knockdown inhibits proliferation and migration of PDAC cells

**Figure 2:**
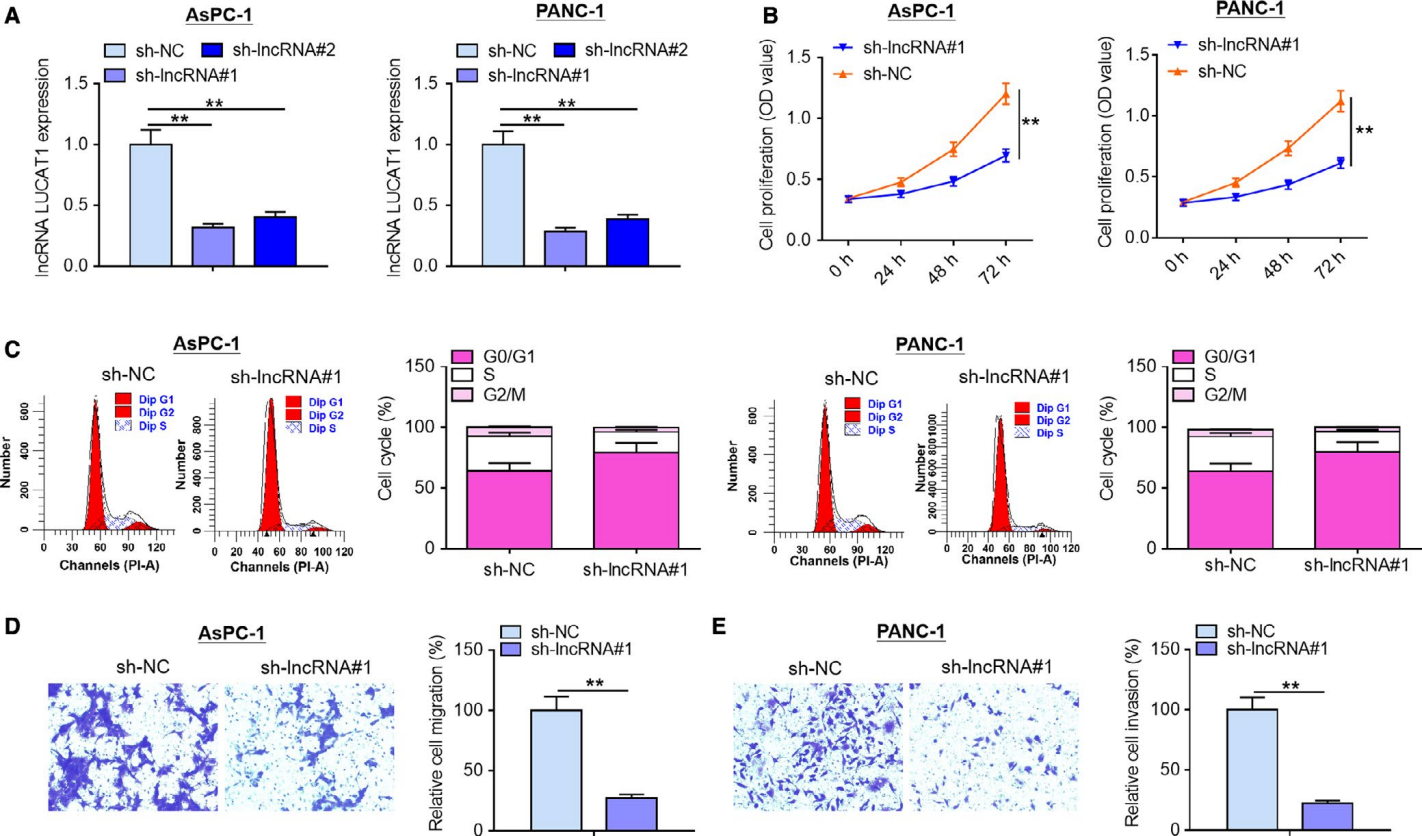
LncRNA LUCAT1 knockdown inhibits proliferation and migration of PDAC cells. (A) PANC‐1 cells and AsPC‐1 cells were transfected with sh‐NC, sh‐lncRNA#1 or sh‐lncRNA#2, followed by qRT‐PCR analysis to assess transfection efficiency. (B) MTT assays were performed to measure proliferation of PANC‐1 cells and AsPC‐1 cells following transfection with sh‐NC or sh‐lncRNA#1. (C) Flow cytometry was carried out to evaluate cell cycle of PANC‐1 cells and AsPC‐1 cells following transfection with sh‐NC or sh‐lncRNA#1. (D) Migration capability and (E) invasion capability of AsPC‐1 and PANC‐1 cells were examined by Transwell migration assays and Transwell invasion assays, respectively. **P < *.*01. lncRNA LUCAT1, long non‐coding RNA lung cancer associated transcript 1; sh‐NC, negative control short hairpin RNA; sh‐lncRNA#1, short hairpin RNA#1 specifically targeting lncRNA LUCAT1; sh‐lncRNA#2, short hairpin RNA#2 specifically targeting lncRNA LUCAT1; qRT‐PCR, quantitative real‐time polymerase chain reaction

In view of the data mentioned above, we speculated that lncRNA LUCAT1 may act as a crucial regulator in PDAC development. To explore the biological functions of lncRNA LUCAT1 in PDAC, we treated AsPC‐1 and PANC‐1 cells, two PDAC cell lines with high endogenous lncRNA LUCAT1 expression, with lncRNA LUCAT1‐specific shRNA#1 (sh‐lncRNA#1), lncRNA LUCAT1‐specific shRNA#2 (sh‐lncRNA#2) and nonspecific negative control RNA (sh‐NC), respectively. As displayed in Figure 2A, lncRNA LUCAT1 expression was dramatically decreased by sh‐lncRNA#1 and sh‐lncRNA#2 in comparison with negative control treatment, especially by sh‐lncRNA#1. Subsequently, sh‐lncRNA#1 was chosen for further studies. As evident from MTT assays, lncRNA LUCAT1 knockdown significantly inhibited AsPC‐1 and PANC‐1 cell proliferation compared with negative control group (Figure 2B). Flow cytometry analysis showed that lncRNA LUCAT1 ablation induced cell cycle arrest in normal cells (Figure 2C). A significant decrease in migration capability of AsPC‐1 and PANC‐1 cells was observed in lncRNA LUCAT1 knockdown group compared with that in negative control group (Figure 2D). Furthermore, invasion ability of normal cells was considerably reduced by lncRNA LUCAT1 depletion compared with negative control treatment (Figure 2E). These results suggest that lncRNA LUCAT1 knockdown inhibits PDAC cell proliferation, migration, and invasion in vitro.

### 3.3 LncRNA LUCAT1 ablation suppresses tumorigenicity of PDAC cells in mouse xenograft models

**Figure 3:**
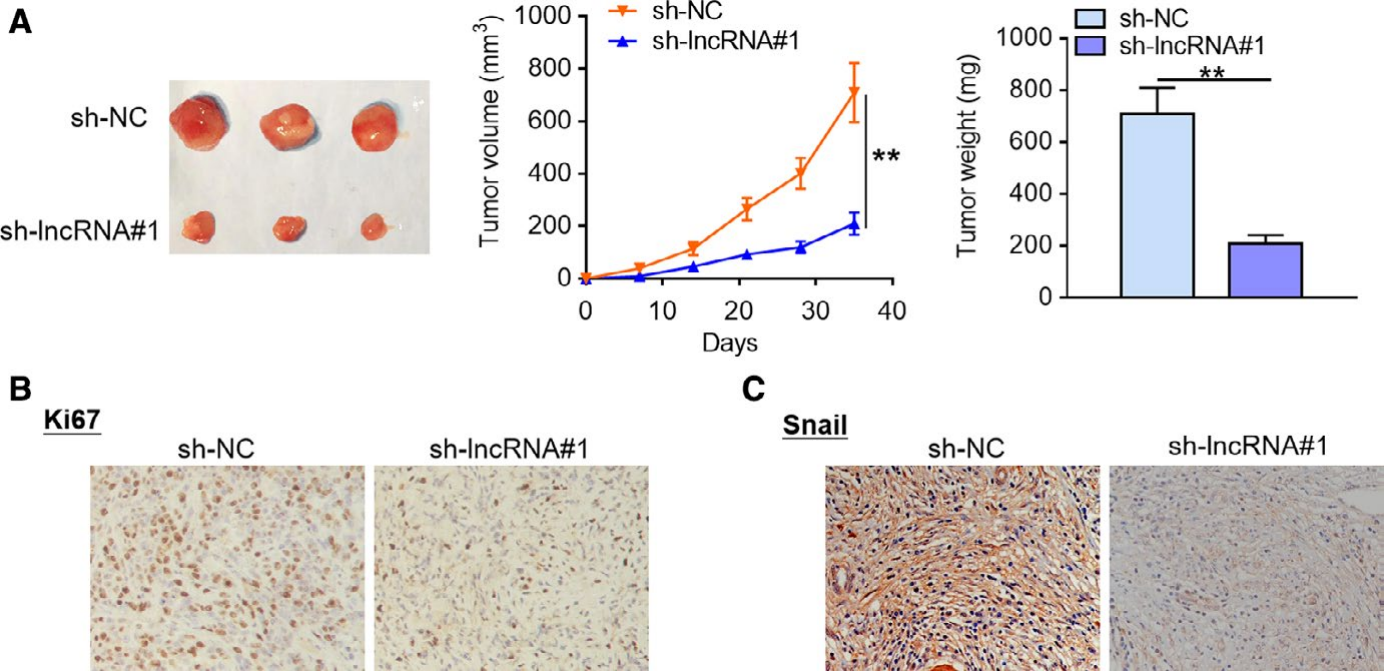
LncRNA LUCAT1 ablation suppresses tumorigenicity of PDAC cells in mouse xenograft models. (A) Tumor xenograft models were established via subcutaneous inoculation of sh‐NC or sh‐lncRNA#1‐treated PANC‐1 cells in the flanks of the nude mice (n = 3). Tumor length and width was measured using a slide caliper every 7 days. The nude mice were sacrificed at day 35 post‐implantation, then tumors were removed and weighed. (B) Ki67 protein expression pattern in the harvested tumor tissues was visualized by IHC staining. (C) Snail protein expression pattern in the collected tumor tissues was characterized using IHC analysis. **P < *.*01. lncRNA LUCAT1, long non‐coding RNA lung cancer associated transcript 1; sh‐NC, negative control short hairpin RNA; sh‐lncRNA#1, short hairpin RNA#1 specifically targeting lncRNA LUCAT1; sh‐lncRNA#2, short hairpin RNA#2 specifically targeting lncRNA LUCAT1

To further evaluate the effects of lncRNA LUCAT1 in vivo, we constructed tumor xenograft models by subcutaneous inoculation of sh‐lncRNA#1‐treated PANC‐1 cells in the flanks of the nude mice. As presented in Figure 3A, a remarkable decrease in tumor weight and volume was observed in the lncRNA LUCAT1 knockdown group in comparison with negative control at day 21 post‐implantation. Furthermore, less Ki67‐positive cells were observed in the tumors collected from the lncRNA LUCAT1 knockdown group compared with those in negative control group (Figure 3B). In addition, tumors formed by sh‐lncRNA#1‐treated PANC‐1 cells exhibited lower Snail expression than those harvested from negative control group (Figure 3C). To sum up, these findings imply that lncRNA LUCAT1 depletion inhibits tumor growth in vivo.

### 3.4 LncRNA LUCAT1 is a molecular sponge of miR‐539

**Figure 4:**
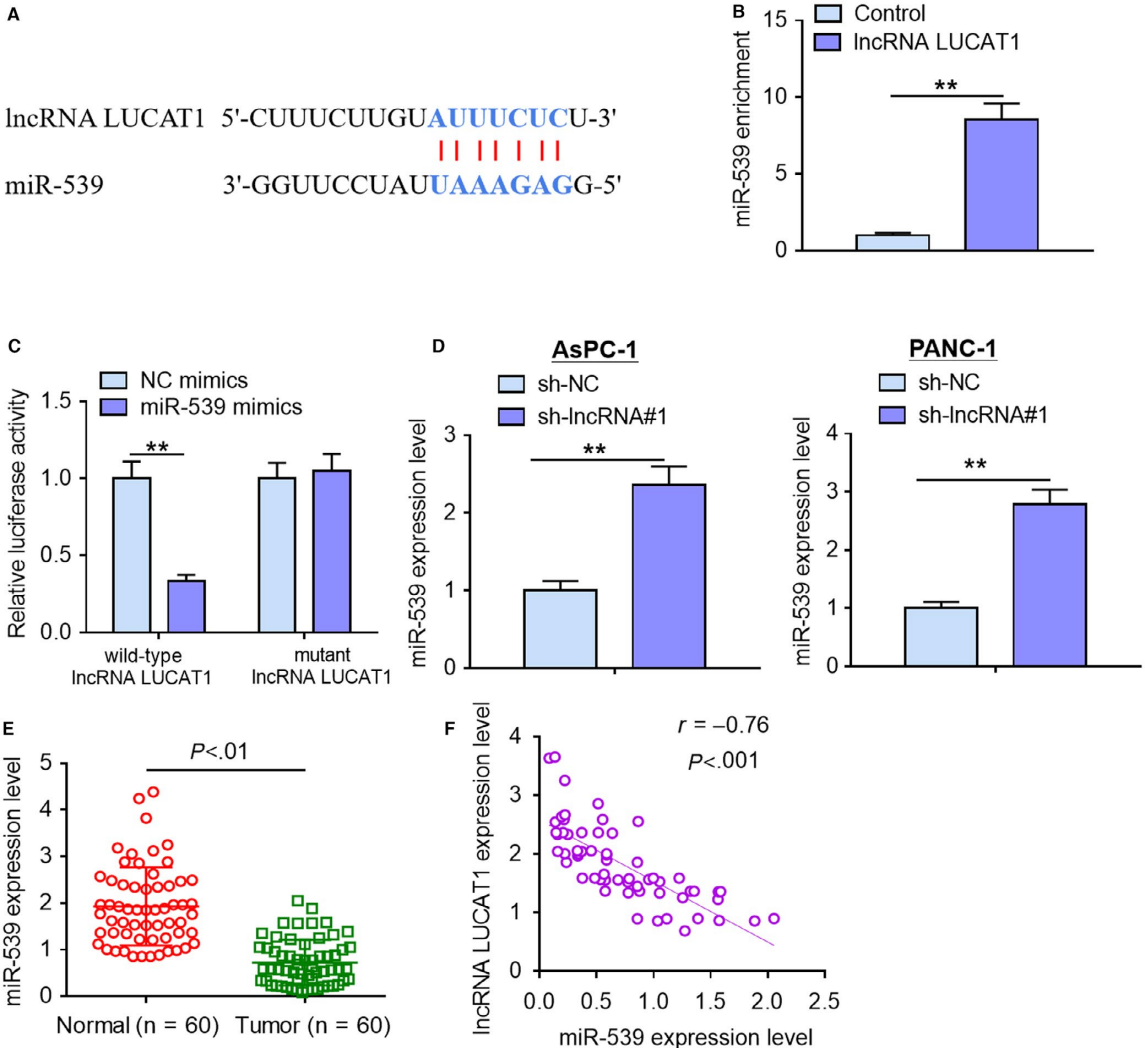
LncRNA LUCAT1 is a molecular sponge of miR‐539. (A) A complementary binding site of lncRNA LUCAT1 in miR‐539 was predicted via starBase v2.0 online database. (B) RIP assays were conducted to validate whether lncRNA could interact with miR‐539 in HEK293T cells. (C) Luciferase reporter assays were carried out to verify the direct binding of lncRNA LUCAT1 and miR‐539 in HEK293T cells. (D) miR‐539 expression levels were detected by qRT‐PCR analysis after transfection with sh‐NC or sh‐lncRNA#1 in AsPC‐1 and PANC‐1 cells. (E) miR‐539 expression levels in tumorous tissues and normal cerebellar tissues were examined using qRT‐PCR analysis. (F) Spearman's correlation analysis was performed to evaluate the relationship between lncRNA LUCAT1 expression and miR‐539 expression in cancerous tissues. **P < *.*01. lncRNA LUCAT1, long non‐coding RNA lung cancer associated transcript 1; sh‐NC, negative control short hairpin RNA; sh‐lncRNA#1, short hairpin RNA#1 specifically targeting lncRNA LUCAT1; sh‐lncRNA#2, short hairpin RNA#2 specifically targeting lncRNA LUCAT1

To illuminate the potential molecular mechanisms by which lncRNA LUCAT1 exerts its functions, we used starBase v2.0 online software to perform bio‐informatics analysis. Growing evidence has confirmed that lncRNAs may exert their roles through sponging their target miRNA. As displayed in Figure 4A, miR‐539 possessed a potential complementary binding site for lncRNA LUCAT1 and was selected as a candidate target for lncRNA LUCAT1. RIP assays demonstrated that more miR‐539 was enriched by lncRNA LUCAT1 over‐expression treatment compared with control treatment, suggesting that lncRNA LUCAT1 could interact with miR‐539 (Figure 4B). To validate the direct binding of lncRNA LUCAT1 and miR‐539, luciferase reporter assays were carried out. As evident from Figure 4C, transfection of miR‐539 mimics led to a significant decrease in luciferase activity of the reporter vectors carrying wild‐type lncRNA LUCAT1, but no significant alterations in luciferase activity were found in mutant groups. qRT‐PCR analysis revealed that miR‐539 expression was remarkably increased by lncRNA LUCAT1 knockdown treatment compared with negative control group (Figure 4D). Moreover tumorous tissues exhibited lower miR‐539 expression than normal tissues (Figure 4E). Notably, Spearman's correlation analysis revealed a significant negative correlation between lncRNA LUCAT1 expression and miR‐539 expression in cancerous tissues (Figure 4F). Collectively, these results indicate that lncRNA LUCAT1 acts as a molecular sponge of miR‐539.

### 3.5 miR‐539 inhibitor reverses the inhibitory effects of lncRNA LUCAT1 knockdown on PDAC cell proliferation and migration

**Figure 5:**
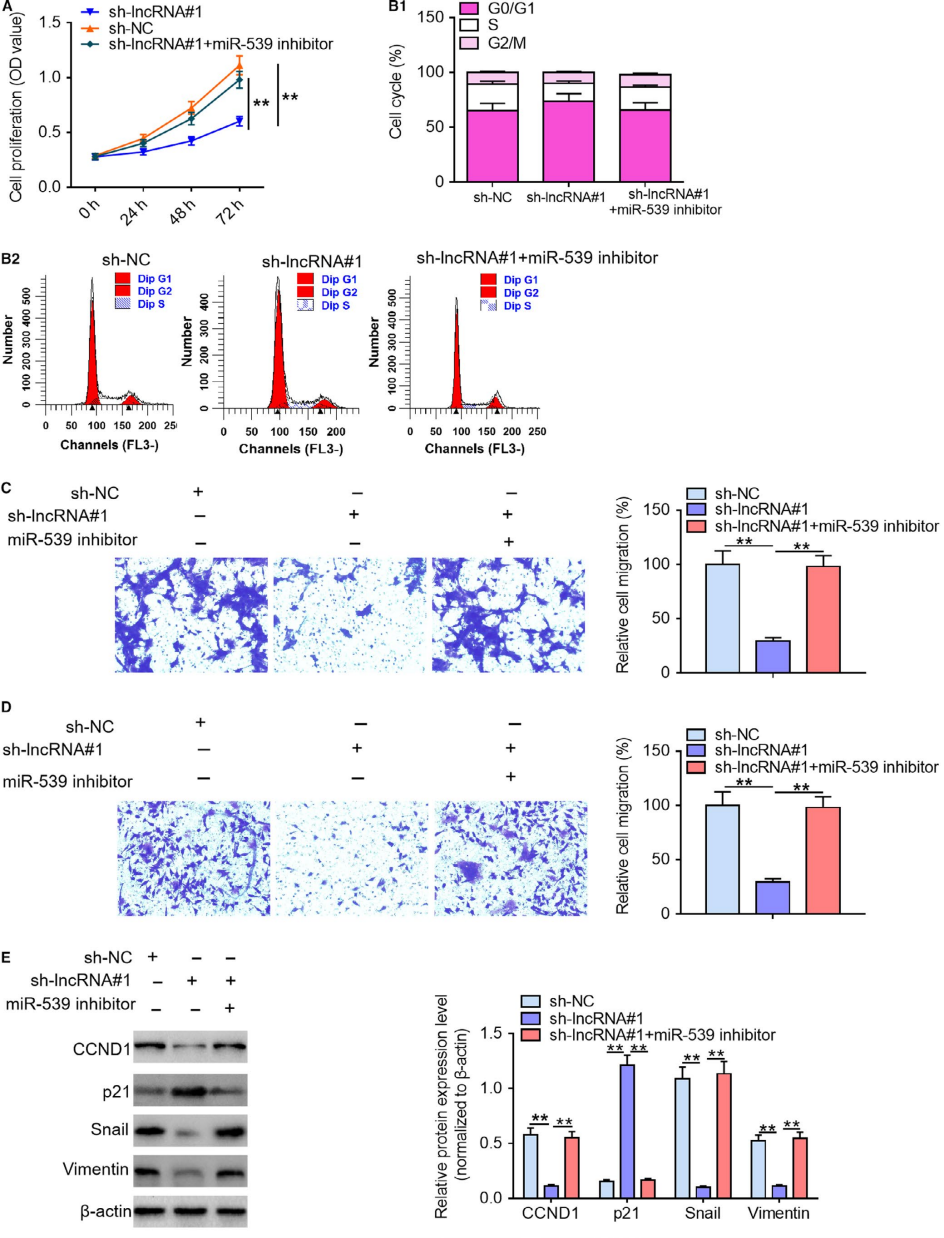
miR‐539 inhibitor reverses the suppressing effects of lncRNA LUCAT1 knockdown on PDAC cell proliferation and migration. (A) Proliferation, (B) cell cycle progression, (C) migration ability, and (D) invasion ability of PANC‐1 cells were determined following transfection with sh‐lncRNA#1 and miR‐539 inhibitor by MTT assays, flow cytometry, and Transwell migration and invasion assays, respectively. (E) The expression levels of cell proliferation‐related proteins (CCND1 and p21) and cell motility‐associated proteins (Snail and Vimentin) in PANC‐1 cells were examined by western blotting assays following transfection with sh‐lncRNA#1 and miR‐539 inhibitor. **P < *.*01. lncRNA LUCAT1, long noncoding RNA lung cancer associated transcript 1; sh‐NC, negative control short hairpin RNA; sh‐lncRNA#1, short hairpin RNA#1 specifically targeting lncRNA LUCAT1; sh‐lncRNA#2, short hairpin RNA#2 specifically targeting lncRNA LUCAT1; CCND1, cyclin D1

To validate the functional correction between lncRNA LUCAT1 and miR‐539, we transfected sh‐lncRNA#1‐treated PANC‐1 cells with miR‐539 inhibitor. Subsequently, we evaluated the effects of miR‐539 down‐regulation on the malignant phenotypes of sh‐lncRNA#1‐treated PANC‐1 cells, including proliferation, cell cycle progression, migration and invasion. As exhibited in Figure 5A and B co‐transfection of sh‐lncRNA#1 and miR‐539 inhibitor considerably facilitated PANC‐1 cell proliferation and cell cycle progression compared with sh‐lncRNA#1 treatment group; moreover, no significant differences were observed between sh‐lncRNA#1 + miR‐539 inhibitor group and negative control group. Furthermore, miR‐539 inhibitor reversed the suppressive effects of lncRNA LUCAT1 ablation on PANC‐1 cell migration and invasion (Figure 5C and D). In addition, western blotting analysis demonstrated that lncRNA LUCAT1 depletion dramatically elevated p21 expression level and reduced CCND1, Snail and Vimentin expression levels in comparison with negative control treatment; notably, miR‐539 down‐regulation reversed the effects of lncRNA LUCAT1 knockdown on CCND1, p21, Snail and Vimentin protein expression (Figure 5E). Collectively, our results manifest that miR‐539 mediates the effects of lncRNA LUCAT1 on malignant phenotypes of PDAC cells.

## 4 DISCUSSION

PDAC, a devastating malignancy, is the most common pathological type of pancreatic cancer and has imposed enormous pressures on public health.^4^, ^26^ Even though some progress has been achieved in the diagnosis and therapy, the prognosis of PDAC patients is still depressing. In consideration of the limitations in conventional treatments, it is imperative to seek novel and effective therapeutic strategies against PDAC. LncRNAs, a novel class of noncoding RNA transcripts, have been identified to play pivotal roles in the development of diverse types of human malignancies.^27^, ^28^ Accumulating evidence has highlighted the biological roles of lncRNAs in the initiation and malignant progression of human PDAC. Yao *et al* found that lncRNA SPRY4‐IT1 was significantly up‐regulated in PDAC tissue samples and contributed to PDAC cell proliferation and survival.^29^ High expression of lncRNA SNHG1 was demonstrated to facilitate PDAC cell proliferation, cell cycle progression, and migration through PI3K/AKT signaling pathway.^30^ It was reported that FEZF1‐AS1 expression was significantly increased in PDAC tissues and that promoted cell growth, invasion and Warburg effect in PDAC.^31^ Previous studies have identified that lncRNA LUCAT1 functions as a vital player in multiple types of human tumors. Zhang *et al* reported that lncRNA LUCAT1 promoted cervical cancer cell proliferation and metastasis via sponging miR‐181a.^20^ Xiao *et al* demonstrated that lncRNA LUCAT1 was a poor prognostic factor and that promoted cell proliferation in vitro and in vivo in renal cell carcinoma.^22^ Kong *et al* displayed that lncRNA LUCAT1 promoted growth, migration and invasion of oral squamous cell carcinoma through regulation of PCNA.^32^ LncRNA LUCAT1 was found to promote tumorigenesis in colorectal cancer through binding with UBA52.^33^ Nonetheless, the biological role of lncRNA LUCAT1 in PDAC remains largely unknown. To characterize the role of lncRNA LUCAT1 in PDAC, we initially examined its expression patterns in clinical tissue samples and PDAC cell lines. Here we found that lncRNA LUCAT1 expression levels were remarkably elevated in both PDAC tissues and cell lines. Notably, increased lncRNA LUCAT1 expression was linked to some clinicopathological characteristics, including larger tumor size and lymphatic invasion. These findings imply that lncRNA LUCAT1 may be implicated in the malignant development of PDAC.

To better understand the biological role of lncRNA LUCAT1 in PDAC, we performed loss‐of‐function studies and evaluated the effects of lncRNA LUCAT1 knockdown on cell proliferation, cell cycle progression, cell migration and invasion. Functional studies displayed that lncRNA LUCAT1 ablation dramatically inhibited cell proliferation, induced cell cycle arrest and considerably retarded cell migration and invasion. Besides, lncRNA LUCAT1 depletion was found to repress tumorigenecity of PDAC cells in nude mouse transplantation models. Altogether, these results indicate that lncRNA LUCAT1 may act as an oncogene in PDAC. Evidence is accumulating that lncRNAs function as endogenous molecular sponges of miRNAs to exert their biological roles. To clarify the potential molecular mechanisms underlying the oncogenic role of lncRNA LUCAT1 in PDAC, mechanistic investigations were conducted. Bio‐informatics analysis, RIP assays and luciferase reporter assays revealed that lncRNA LUCAT1 was a molecular sponge of miR‐539. Furthermore, rescue experiments validated that miR‐539 mediated the effects of lncRNA LUCAT1 on malignant phenotypes of PDAC cells. Past studies have demonstrated that miR‐539 acts as a tumor suppressor in nervous system neoplasms and other human malignancies, including glioma,^34^ meningioma,^35^ osteosarcoma,^36^ hepatocellular carcinoma,^37^ prostate cancer^38^ and triple‐negative breast cancer.^39^ It is well documented that decreased miR‐539 expression is associated with uncontrolled proliferation and malignant metastasis of cancer cells.^36^, ^38^, ^40^, ^41^

In conclusion, this study for the first time highlighted the oncogenic role of lncRNA LUCAT1 in PDAC, including facilitating cell proliferation, cell cycle progression, migration and invasion. Furthermore, our data revealed that lncRNA LUCAT1 exerted its carcinogenic functions in PDAC through sponging miR‐539. Collectively, our findings indicate that targeting lncRNA LUCAT1/miR‐539 axis may be used as a candidate therapeutic strategy against PDAC.

## CONFLICT OF INTEREST

The authors declare that no conflicts of interest exist in this study.

## AUTHOR CONTRIBUTIONS

Yong Ma and Yongjun Nai conceived and designed this study. Yongjun Nai and Chao Pan conducted relevant experiments, performed statistical analysis, and manuscript preparation. Xueteng Hu participated in data analysis and manuscript preparation. All the authors read and approved the submission of this work.
